# Supplementary material for: Two Bacillus spp. Strains Improve the Structure and Diversity of the Rhizosphere Soil Microbial Community of Lilium brownii var. viridulum
Source: Microorganisms. 2023 May 6;11(5):1229. doi: 10.3390/microorganisms11051229 (PMC10223157; doi:10.3390/microorganisms11051229)
Supplement: Supplementary file 1 [file microorganisms-11-01229-s001.zip › microorganisms-2325728-SI.pdf]

Table S1 Relative abundance of fungal community structure in lily rhizosphere soil samples (at the phylum level).

Phylum	BG (%)	CK (%)	BF1 (%)	Y37 (%)
Ascomycota	31.21 ± 15.43a	39.97 ± 12.32a	44.77 ± 14.22a	44.02 ± 6.53a
Basidiomycota	9.75 ± 8.65a	7.45 ± 2.60a	5.13 ± 0.83a	5.71 ± 1.44a
Rozellomycota	1.88 ± 1.15a	6.71 ± 8.69a	0.30 ± 0.20a	0.54 ± 0.60a
Mortierellomycota	1.01 ± 0.96b	2.11 ± 2.50b	2.73 ± 1.23ab	5.67 ± 3.11a
Chytridiomycota	0.96 ± 0.39a	4.79 ± 4.85a	3.32 ± 5.28a	0.97 ± 0.85a
Glomeromycota	1.21 ± 0.05a	1.26 ± 0.15a	1.34 ± 0.44a	0.99 ± 0.16a
Kickxellomycota	1.03 ± 2.79a	0.80 ± 0.15a	1.40 ± 0.06a	0.86 ± 0.28a
Mucoromycota	0.16 ± 0.01a	0.39 ± 0.06a	0.18 ± 0.09a	0.23 ± 0.01a
Monoblepharomycota	0.00 ± 0.00a	0.01 ± 0.02a	0.01 ± 0.01a	0.01 ± 0.01a
Olpidiomycota	0.00 ± 0.00a	0.04 ± 0.05a	0.01 ± 0.01a	0.03 ± 0.05a
Zoopagomycota	0.00 ± 0.00a	0.01 ± 0.01a	0.00 ± 0.00a	0.02 ± 0.03a
Entomophthoromycota	0.00 ± 0.00a	0.00 ± 0.00a	0.00 ± 0.00a	0.01 ± 0.01a
Others	52.81 ± 12.78a	38.36 ± 10.85a	43.44 ± 14.68a	42.60 ± 7.56a

Table S2 Relative abundance of fungal community structure in lily rhizosphere soil samples (top 20 at the genus level).

Genus	BG (%)	CK (%)	BF1 (%)	Y37 (%)
Colletotrichum	2.27 ± 3.22a	8.10 ± 2.49a	11.94 ± 10.90a	8.47 ± 5.65a
Humicola	1.95 ± 1.30a	1.77 ± 0.79a	2.38 ± 0.69a	7.39 ± 10.22a
Fusarium	3.00 ± 2.99a	2.31 ± 0.61a	2.89 ± 1.25a	1.95 ± 0.84a
Solicoccozyma	0.41 ± 0.34b	2.37 ± 1.31a	2.46 ± 0.84a	2.72 ± 0.75a
Talarmyces	0.21 ± 0.17b	0.96 ± 1.00b	0.90 ± 0.85b	5.18 ± 3.15a
Penicillium	1.66 ± 0.88a	0.90 ± 1.11a	1.15 ± 0.66a	2.60 ± 3.68a
Myceliophthora	0.41 ± 0.37b	0.91 ± 0.23ab	1.30 ± 0.34a	1.11 ± 0.65a
Conocybe	3.65 ± 7.23a	0.00 ± 0.00a	0.00 ± 0.00a	0.00 ± 0.00a
Saitozyma	0.23 ± 0.18b	1.39 ± 0.40a	0.63 ± 0.39ab	1.28 ± 0.74a
Chaetomium	0.88 ± 0.88a	0.47 ± 0.13a	0.94 ± 0.45a	1.15 ± 0.44a
Gymnopilus	2.59 ± 4.42a	0.04 ± 0.05a	0.03 ± 0.03a	0.16 ± 0.12a
Curvularia	0.31 ± 0.52a	0.56 ± 0.34a	0.39 ± 0.34a	0.93 ± 0.57a
Echria	0.25 ± 0.38a	0.43 ± 0.48a	0.10 ± 0.13a	1.02 ± 1.88a
Arthrobotrys	0.02 ± 0.03b	0.05 ± 0.04b	1.54 ± 1.65a	0.02 ± 0.02b
Ophiocordyceps	0.88 ± 0.85a	0.72 ± 1.38a	0.00 ± 0.00a	0.03 ± 0.02a
Polyschema	1.07 ± 1.14a	0.15 ± 0.14b	0.09 ± 0.12b	0.04 ± 0.03b
Alternaria	0.61 ± 0.71a	0.24 ± 0.05a	0.12 ± 0.08a	0.31 ± 0.09a
Nephroma	0.76 ± 1.40a	0.20 ± 0.15a	0.08 ± 0.04a	0.16 ± 0.13a
Trichosporon	0.29 ± 0.32a	0.37 ± 0.41a	0.40 ± 0.65a	0.14 ± 0.23a
Gibellulopsis	0.18 ± 0.33a	0.09 ± 0.04a	0.38 ± 0.53a	0.20 ± 0.22a

Table S3 Relative abundance of bacterial community structure in lily rhizosphere soil samples (top 10 at the phylum level).

Phylum	BG (%)	CK (%)	BF1 (%)	Y37 (%)
Proteobacteria	36.79 ± 10.87a	35.23 ± 9.10a	40.75 ± 3.20a	34.52 ± 4.72a
Acidobacteria	35.12 ± 11.90a	30.53 ± 7.87ab	19.98 ± 5.71b	30.79 ± 5.15ab
Bacteroidetes	4.09 ± 1.74a	3.30 ± 1.03a	3.08 ± 0.73a	3.27 ± 0.76a
Chloroflexi	12.31 ± 3.96a	8.57 ± 3.50a	8.64 ± 5.05a	9.54 ± 3.11a
Actinobacteria	4.02 ± 1.10c	7.08 ± 1.82b	11.81 ± 1.56a	8.95 ± 1.70b
Gemmatimonadetes	0.93 ± 0.31b	3.73 ± 1.60ab	5.34 ± 3.39a	2.32 ± 1.28ab
Nitrospirae	0.49 ± 0.25b	1.91 ± 1.42a	1.31 ± 0.81ab	1.18 ± 0.34ab
Verrucomicrobia	1.38 ± 0.73b	2.43 ± 0.95a	0.94 ± 0.34b	1.28 ± 0.33b
Firmicutes	0.60 ± 0.75ab	0.36 ± 0.09b	1.38 ± 0.82a	0.89 ± 0.08ab
Unidentified_Bacteria	0.78 ± 0.08a	1.04 ± 0.44a	0.67 ± 0.22a	0.66 ± 0.24a

Table S4 Relative abundance of bacterial community structure in lily rhizosphere soil samples (top 20 at the genus level).

Genus	BG (%)	CK (%)	BF1 (%)	Y37 (%)
Bryobacter	19.14 ± 9.15a	4.44 ± 1.74b	3.49 ± 1.22b	3.69 ± 0.46b
Chujaibacter	15.91 ± 12.49a	1.32 ± 0.47b	0.93 ± 0.56b	1.17 ± 0.63b
Unidentified_Burkholderiaceae	0.34 ± 0.28b	1.90 ± 1.16b	4.62 ± 2.28a	5.21 ± 1.82a
Sphingomonas	1.21 ± 0.25b	2.30 ± 1.13ab	3.96 ± 1.43a	3.13 ± 1.03a
Candidatus_Solibacter	1.01 ± 0.26b	3.42 ± 1.71a	1.76 ± 0.38b	2.41 ± 0.69ab
Acidibacter	1.30 ± 1.03a	1.94 ± 0.37a	1.86 ± 0.74a	1.79 ± 0.83a
Gemmatimonas	0.44 ± 0.20a	2.27 ± 1.43a	2.31 ± 2.49a	0.84 ± 0.57a
Bradyrhizobium	0.27 ± 0.14b	0.87 ± 0.37b	2.24 ± 1.22a	2.40 ± 0.26a
Unidentified_Acidimicrobiia	1.19 ± 0.83a	1.60 ± 0.65a	1.41 ± 0.64a	1.51 ± 0.61a
Rhodanobacter	1.09 ± 0.53b	0.71 ± 0.23b	2.06 ± 0.91a	0.95 ± 0.15b
Unidentified_Gammaproteobacteria	0.56 ± 0.15b	1.01 ± 0.17ab	1.70 ± 0.57a	1.14 ± 0.65ab
Haliangium	0.56 ± 0.39a	1.61 ± 0.82a	1.10 ± 0.72a	0.92 ± 0.78a
Pseudolabrys	0.47 ± 0.25b	2.08 ± 1.15a	0.93 ± 0.33b	0.67 ± 0.33b
Granulicella	0.47 ± 0.35b	1.03 ± 0.12ab	1.01 ± 0.33ab	1.13 ± 0.61a
Acidothermus	0.50 ± 0.19b	0.42 ± 0.17b	1.41 ± 0.35a	0.78 ± 0.26b
unidentified_Bacteria	0.75 ± 0.07a	1.01 ± 0.44a	0.65 ± 0.22a	0.62 ± 0.21a
Terracidiphilus	0.24 ± 0.04b	0.51 ± 0.15b	0.38 ± 0.12b	1.18 ± 0.62a
unidentified_Verrucomicrobiae	0.71 ± 0.57ab	0.93 ± 0.56a	0.14 ± 0.05b	0.29 ± 0.15ab
Acidicaldus	0.72 ± 0.24a	0.47 ± 0.18ab	0.56 ± 0.50ab	0.22 ± 0.06b
Nitrosospira	0.08 ± 0.05a	0.54 ± 0.29a	0.59 ± 0.17a	0.74 ± 0.95a
